# Handoffs and the challenges to implementing teamwork training in the perioperative environment

**DOI:** 10.3389/fpsyg.2023.1187262

**Published:** 2023-06-15

**Authors:** Shannon Paquette, Molly Kilcullen, Olivia Hoffman, Jessica Hernandez, Ankeeta Mehta, Eduardo Salas, Philip E. Greilich

**Affiliations:** ^1^Office of Undergraduate Medical Education, UT Southwestern Medical Center, Dallas, TX, United States; ^2^Department of Psychological Sciences, Rice University, Houston, TX, United States; ^3^Division of Critical Care Medicine, Department of Pediatrics, UT Southwestern Medical Center, Dallas, TX, United States; ^4^Department of Emergency Medicine, UT Southwestern Medical Center, Dallas, TX, United States; ^5^Department of Surgery, UT Southwestern Medical Center, Dallas, TX, United States; ^6^Department of Anesthesiology and Pain Management, Health System Chief Quality Office, Office of Undergraduate Medical Education, UT Southwestern Medical Center, Dallas, TX, United States

**Keywords:** teamwork, team training, interprofessional, handoffs, perioperative, healthcare education, implemenation, patient safety

## Abstract

Perioperative handoffs are high-risk events for miscommunications and poor care coordination, which cause patient harm. Extensive research and several interventions have sought to overcome the challenges to perioperative handoff quality and safety, but few efforts have focused on teamwork training. Evidence shows that team training decreases surgical morbidity and mortality, and there remains a significant opportunity to implement teamwork training in the perioperative environment. Current perioperative handoff interventions face significant difficulty with adherence which raises concerns about the sustainability of their impact. In this perspective article, we explain why teamwork is critical to safe and reliable perioperative handoffs and discuss implementation challenges to the five core components of teamwork training programs in the perioperative environment. We outline evidence-based best practices imperative for training success and acknowledge the obstacles to implementing those best practices. Explicitly identifying and discussing these obstacles is critical to designing and implementing teamwork training programs fit for the perioperative environment. Teamwork training will equip providers with the foundational teamwork competencies needed to effectively participate in handoffs and utilize handoff interventions. This will improve team effectiveness, adherence to current perioperative handoff interventions, and ultimately, patient safety.

## Introduction

Patient handoffs are “real-time processes of passing patient-specific information from one caregiver to another or from one team of caregivers to another for the purpose of ensuring the continuity of the patient’s care” (The Joint Commission, 2017). Regulating bodies that oversee medical education recognize the importance of handoffs; the Accreditation Council for Graduate Medical Education (ACGME) outlines requirements for transitions-in-care training during residency, and the Association of American Medical Colleges identifies patient handovers as an Entrustable Professional Activity that all medical students should be able to perform before residency (Obeso et al., 2017; ACGME, 2020). Hospital and patient safety organizations consider handoffs high-risk events for communication errors, contributing to sentinel events and significant malpractice costs (The Joint Commission, 2017; Humphrey et al., 2022). The perioperative environment is particularly vulnerable: a single operation requires at least two interprofessional handoffs—preoperative and postoperative—and many surgeries also require several intraoperative handoffs (Frasier et al., 2020; Meersch et al., 2022).

Teamwork skills are essential to addressing perioperative handoff quality and safety issues and delivering safe patient care. Evidence shows that teamwork improves patient, staff, and healthcare organizational outcomes (Rosen et al., 2018). Furthermore, meta-analytic evidence indicates that teamwork significantly impacts healthcare team performance (Schmutz et al., 2019). Recent conceptual models have illustrated that teamwork competencies are imperative to handoff effectiveness (Webster et al., 2022), and meta-analytic evidence shows that teamwork training significantly impacts reactions, learning, transfer, and results across healthcare contexts (i.e., organizational and patient outcomes; Hughes et al., 2016). In the perioperative environment, less frequent application of teamwork skills, such as sharing unique information and briefing, has been associated with increased complications and mortality (Segall et al., 2012). Additional meta-analytic evidence indicates that communication tools (e.g., checklists) improve teamwork and reduce mortality and morbidity in surgical contexts (Lyons and Popejoy, 2014). To improve organizational and patient outcomes in the perioperative realm, where teams are dynamic and patients experience frequent interprofessional handoffs during a high-risk, high-acuity, and high-pressure period, providers must demonstrate adaptability and excellence in teamwork competencies (Segall et al., 2012; Webster et al., 2022). Team science experts recommend teamwork training for medical teams to increase their adaptability to non-routine events (Bedwell et al., 2012), and this advice is particularly relevant for dynamic perioperative teams.

This perspective article discusses the importance of teamwork in perioperative handoffs and challenges to implementing teamwork training in this environment. We propose that teamwork training will improve team effectiveness, adherence to perioperative handoff interventions, and, ultimately, patient safety. Our article provides a foundation for improving teamwork training in perioperative contexts by outlining evidence-based best practices imperative for training success, while acknowledging obstacles to implementing those best practices. We assert that explicitly identifying and discussing these obstacles will provide a critical resource for designing and delivering teamwork training programs fit for the perioperative environment.

## Importance of teamwork in interprofessional perioperative handoffs

Perioperative handoffs are particularly challenging due to this setting’s unique interdependence of interprofessional roles, acuity and complexity of care, handoff frequency, time constraints, and environmental distractions (Etherington et al., 2019; Abraham et al., 2021b; Michael et al., 2021). Previous interventions have improved perioperative handoff quality; however, high-quality studies demonstrating improved patient outcomes are still needed (Lyons and Popejoy, 2014; Abraham et al., 2021c,d; Stenquist et al., 2022). Furthermore, providers have difficulty adhering to perioperative handoff interventions due to time constraints, competing priorities, and the low perceived utility of these tools, which raises concerns about their sustainability (Abraham et al., 2021a; Burden et al., 2021). Providers need to be trained to effectively utilize such tools and appreciate their importance. For example, providers must learn to effectively employ skills such as structured and closed-loop communication, and asking clarifying questions to get the most out of a mnemonic handoff tool (Greilich et al., 2023). Perioperative team training has been associated with improved teamwork behaviors as well as decreased surgical morbidity and mortality (Neily et al., 2010; Weaver et al., 2010; Rhee et al., 2017). Despite this evidence, there remains a widespread lack of sustained team training in the perioperative space. A review by Raveendran et al. (2023) noted that most current interventions address only a few teamwork constructs and called for perioperative training programs that comprehensively address teamwork competencies and measure interprofessional outcomes. In response to The Joint Commission’s (2017) sentinel event alert, the Anesthesia Patient Safety Foundation created guidelines for the execution and research of perioperative handoffs, concluding that teamwork training and attitude/behavior changes are essential for successful perioperative handoff interventions (Agarwala et al., 2019). Teaching teamwork competencies will foster the attitudes and behaviors needed to improve provider adherence to these interventions.

The perioperative space has a unique amalgamation of interprofessional roles working together to deliver care. The variety of professional identities, communication styles, educational backgrounds, competing priorities, and perceived hierarchies strain interprofessional teamwork (Etherington et al., 2019). Interprofessional team members contribute information disproportionately, and miscommunications occur more frequently during exchanges between *different* professions (Cumin et al., 2017; Keller et al., 2019). Perceived hierarchies contribute to this unequal information sharing by impacting psychological safety and team trust, which discourages certain members from speaking up (Cumin et al., 2017; Etherington et al., 2019; Keller et al., 2019). Poor uptake of perioperative handoff interventions may also result from limited team member engagement for the entirety of a handoff (Abraham et al., 2021b). Teamwork training will help overcome some of these obstacles by strengthening team members’ foundational teamwork competencies, such as recognizing the criticality of teamwork, creating a psychologically safe environment, establishing mutual trust, and optimizing team mental models to improve engagement (King et al., 2008; Greilich et al., 2023).

The perioperative environment requires many handoffs including pre-, intra-, and postoperative handoffs as well as intra- and interprofessional handoffs. Preoperative intraprofessional handoffs occur between the preoperative nurse and operating room circulating nurse, for example, while preoperative interprofessional handoffs may occur between the preoperative anesthesia team and the operative team. Some postoperative interprofessional examples include the operative team to the anesthesia provider and nurse in the post-anesthesia care unit or surgeon to the advanced practice provider in the intensive care unit (Frasier et al., 2020; Burden et al., 2021; Meersch et al., 2022). These handoffs provide multiple opportunities for poor care coordination which is exacerbated by the detailed information exchange required for perioperative patients. These patients are acutely vulnerable due to the inherent risks of undergoing anesthesia and invasive procedures and the severity of pathologies that merit surgical intervention (Devereaux and Sessler, 2015; Fernandez-Bustamante et al., 2017; Aminian et al., 2022; Talmasov and Klein, 2022). The performance requirements for surgical patient care result in time pressure within an individual patient’s care continuum and interpatient care, as multiple patients require high-level care simultaneously (Etherington et al., 2019; Göras et al., 2019). This time pressure often results in multitasking, which correlates with performance degradation and occurs almost 50% of the time during operative care (Göras et al., 2019; Modi et al., 2020). The high workload conditions created by time pressure causes team members to prioritize their own tasks, decrease attention to other team members’ needs, and disengage from activities that benefit the team and overall patient care (e.g., handoffs) if a direct correlation with their individual responsibilities is not clear (Shaw et al., 2010; Etherington et al., 2019). Furthermore, different interprofessional operative team members experience varying workloads and stress levels at different time points in care (Aouicha et al., 2020). Training providers in requisite teamwork competencies, such as the criticality of teamwork, mutual performance monitoring, debriefing, and mutual trust, will help address issues created by time pressure and care complexity (Greilich et al., 2023).

The aforementioned interprofessional nature, time pressure, and care demands of the perioperative environment produce many opportunities for interruptions and distractions that harm handoffs. Interruptions can range from technical tasks, such as managing equipment, to interpersonal, such as other providers initiating conversations about tasks unrelated to the patient at hand (Etherington et al., 2019; Göras et al., 2019; Aouicha et al., 2020; Frasier et al., 2020; Modi et al., 2020). Previous studies demonstrated that disruptions during perioperative handoffs occur frequently (~45% of the time; Frasier et al., 2020) and sometimes result in team members leaving the handoff, further impeding handoff intervention adherence (Abraham et al., 2021b). Providers equipped with the appropriate teamwork skills, such as closed-loop communication, optimizing team mental models, and reflection/debriefing, are more likely to reduce the frequency and impact of such interruptions, ensuring team effectiveness (Salas et al., 2008; Zajac et al., 2021; Greilich et al., 2023).

## Challenges to implementing impactful teamwork training programs in the perioperative environment

While there is abundant evidence of the effectiveness of teamwork training and interventions in healthcare (Hughes et al., 2016), evidence in the perioperative environment is mixed (Turcotte et al., 2022), indicating potential issues with program implementation. A review by Teunissen et al. (2020) found that perioperative teamwork is not widely understood. Additionally, a systematic review by Turcotte et al. (2022) showed that current interprofessional perioperative interventions have not yet demonstrated improved provider satisfaction. To optimize the impact of team training in the perioperative environment, programs must meaningfully incorporate science-based learning and training best practices. Training transfer literature emphasizes the importance of what happens before, during, and after training. Healthcare organizations frequently focus on factors that occur during training. However, training science tells us that the most important aspects of training are those done *before* and *after* training (Salas et al., 2018). Program developers must consider five critical components that affect training outcomes: facilitator education, trainee composition, training timing, training evaluation, and supportive conditions (see Table 1). These components are resource-intensive and present major obstacles to successful training. Though they generally apply to all environments in need of team training, they are particularly critical in the perioperative realm. Below, we discuss these five components, the challenges to incorporating them, and the unique aspects of these challenges within the perioperative environment.

**Table 1:** Challenges to implementing teamwork training programs in the perioperative environment.

Challenge	Description
1. Providing facilitator-led education	Utilizing a facilitated training method in conjunction with a train-the-trainer approach
2. Coordinating interprofessional training	Training students and practicing professionals together to improve interdisciplinary (e.g., anesthesiologists, intensivists, surgeons) and interprofessional (e.g., nurses, nurse practitioners, physicians, physician assistants, respiratory therapists, surgical technicians) teamwork coordination and communication
3. Training preclinically and longitudinally	Training preclinically with continual refresher trainings to ensure that effective behaviors are learned from the beginning and sustained over time
4. Comprehensively evaluating training impact	Meaningfully evaluating the reactions, learning, transfer, and results of training preclinically, clinically, and post-graduation
5. Creating supportive conditions to sustain behaviors	Establishing institutional and supervisory support (e.g., resources, policies, behavioral modeling) for teamwork training, behaviors, and initiatives

### Challenge 1: Providing facilitator-led education

Successful training programs must include facilitators who are knowledgeable in training content and delivery. Existing best practices advise a train-the-trainer approach to ensure that facilitators successfully deliver the necessary knowledge and skills to trainees. Compared with self-study approaches, train-the-trainer strategies significantly improve provider adherence and competence (e.g., TeamSTEPPS^™^; King et al., 2008; Martino et al., 2011). Facilitators should represent all roles within the team (ex. anesthesiologists, intensivists, nurses, surgeons). However, facilitator-led training demands considerable resources: external facilitators require funding and lack contextual knowledge of the perioperative environment’s intricacies, whereas training internal facilitators delays training onset. Moreover, obtaining protected non-clinical time for perioperative providers to act as internal facilitators is particularly challenging. Teams of interprofessional providers must dedicate time to facilitator training and delivering the curriculum that would otherwise typically be spent teaching technical skills for the operative environment or providing operative services, which are high-value activities for hospitals (Best et al., 2020).

### Challenge 2: Coordinating interprofessional training

Whenever possible, individuals in different yet interdependent interprofessional roles must train together. Interprofessional training increases program fidelity, i.e., the extent to which the simulation (e.g., training) and knowledge and skills learned match the simulated system (e.g., the perioperative environment; Farmer et al., 1999; Maran and Glavin, 2003). Interprofessional team training shows significant improvements in team knowledge, skills, and communication (Nelson et al., 2017). However, incorporating interprofessional training into the perioperative environment requires extensive coordination to balance the competing responsibilities of various roles and ensure that training activities do not disrupt operative services (Etherington et al., 2019; Abraham et al., 2021a). Training content must optimize relevance to all professions without limiting on-the-job context for each role. If the training content does not align with functional tasks, it can devastate transfer of trained behavior to the job, ultimately nullifying the effectiveness and significance of training (Hamstra et al., 2014).

### Challenge 3: Training preclinically and longitudinally

Training best practices indicate it is imperative to consider training timing, specifically regarding career stages (e.g., undergraduate vs. graduate medical education) and the duration of training (e.g., a single workshop vs. progressive or recurring context-specific sessions). Despite the increasing prevalence of teamwork training initiatives in healthcare, standardized implementation of these efforts in healthcare education lags (Weaver et al., 2014; Buljac-Samardzic et al., 2020). Recent reviews substantiate that preclinical teamwork training is limited and call for health education programs to incorporate more teamwork training (Fox et al., 2018; Gordon et al., 2018; Vuurberg et al., 2019).

Individuals should learn teamwork competencies preclinically to establish a common language and appreciation for teamwork before they adopt ineffective team behavioral norms. Training efforts are less effective for established providers because existing knowledge and norms make learning and incorporating new material in practice more difficult (Anderson and Neely, 1996). Providers find unlearning ineffective team behaviors difficult for a multitude of reasons: they may struggle to break existing habits and routines and unlearn mental shortcuts (e.g., mindsets about how teamwork should be conducted), fear the unknown of new norms and their effect on patient safety, and lack awareness about the benefits of unlearning (Rushmer and Davies, 2004). These obstacles are exacerbated by the time pressure of the perioperative environment. However, it can be difficult to incorporate teamwork training into preclinical student course schedules and develop team training curricula applicable to all professions while maintaining training fidelity. Again, the acuity of care and time pressure in the perioperative environment impede the incorporation of critical training activities, like practice and debriefing, into students’ perioperative rotations. Additionally, while training preclinically is essential to improve teamwork capabilities for future providers, it does not address the gap in teamwork skills of practicing professionals. Introducing teamwork education and training at the preclinical level can address this issue by equipping learners with prerequisite skills to engage in on-the-job perioperative team training like NetworkZ and adapted version of TeamSTEPPS for the perioperative environment (Weaver et al., 2010; Rhee et al., 2017; Jowsey et al., 2019).

The intended duration of training is also important. Although few studies have sought the optimal interval for refresher teamwork training (Weaver et al., 2014), the existing literature does indicate that refresher training is needed to sustain teamwork skills in healthcare (Steinemann et al., 2011). A systematic review of teamwork training studies by Marlow et al. (2017) indicated that distributed training sessions can reinforce the importance of teamwork over time. While not focused on teamwork training, a systematic review of training in emergency care by Ameh et al. (2019) revealed that longer training programs were associated with greater skills improvement and asserted that knowledge and skills can be retained for up to a year, but repeat training and opportunities to practice improve retention (Ameh et al., 2019). Other clinical work research shows that knowledge and skills deteriorate as quickly as 3 to 6 months following training, implying that refresher training may be necessary after this duration (Mancini et al., 2010). Longitudinal teamwork training with refresher intervals requires dedicated resources (e.g., time away from practice, funding for facilitators) and coordination between practicing institutions and educational programs to ensure that content aligns with previous coursework. Previous reviews indicate that teamwork training typically occurs in single sessions, indicative of these resource challenges, which have amplified impact in perioperative spaces (Husebø and Akerjordet, 2016; Fox et al., 2018).

### Challenge 4: Comprehensively evaluating training impact

Existing best practices urge incorporation of rigorous evaluation methods to track the effectiveness of training programs. A training program’s fidelity and impact on meaningful behavior changes and relevant outcomes cannot be determined without comprehensive evaluation. Currently, training program evaluations are mostly self-reported with some observational ratings (Fox et al., 2018; Li et al., 2018). These methods have varying degrees of reliability and validity (Li et al., 2018) and fail to capture the true outcomes of teamwork training. A review by Chakraborti et al. (2008) showed that most teamwork training programs failed to track teamwork or outcomes beyond the end of the program. A later systematic review found that only 40% of programs tracked outcomes, although several studies did track the transfer of teamwork skills up to 12 months post-training (Costar and Hall, 2020). Notably, this review included only 20 studies and excluded articles that included medical or nursing students.

The Kirkpatrick evaluation model considers four levels of evaluating training program effectiveness: reactions (trainee satisfaction and perceived utility of the training), learning (the knowledge and skills that trainees gain), transfer (the transfer of learned knowledge and skills to the work environment), and results (the training’s impact on individual, team, and organizational outcomes; Kirkpatrick, 1998). Training programs must be rigorously evaluated on all four levels to claim effectiveness. If data can support positive impacts on all levels, this presents a convincing argument for organizations to expend resources to support the training program. However, effective evaluation of each Kirkpatrick level can be time- and resource-intensive and require dedicated personnel. For example, handoff processes frequently involve electronic medical records, but using them to measure outcomes and skills transfer in the perioperative environment would require significant institutional investment in clinical informatics specialists for development (Abraham et al., 2023). The frequency of perioperative handoffs also obscures each dynamic team’s influence on patient outcomes. If some teams undergo team training but others do not, it is challenging to delineate the training’s impact on outcomes. Therefore, comprehensive training of all interprofessional providers involved in perioperative care is critical for accurate program evaluation.

### Challenge 5: Creating supportive conditions to sustain behaviors

Improving perioperative handoff safety requires an environment conducive to teamwork. Local interventions at the unit level are often insufficient without institutional support, and previous perioperative teamwork interventions and systematic reviews of this work have consistently identified this as a crucial obstacle to program success (Jowsey et al., 2019; Teunissen et al., 2020; Keebler et al., 2022; Turcotte et al., 2022; Raveendran et al., 2023). Team members will continue to encounter challenges that increase errors if institutional structures do not allocate time and resources to conducting safe team-based handoffs. Meta-analytic findings substantiate that a supportive work environment is a critical predictor of learning transfer (Blume et al., 2010).

However, establishing conditions to sustain trained behaviors presents several challenges. Changing work culture is difficult and requires time and resources. Recent literature indicates that healthcare organizations must implement a multi-level approach, incorporating both top-down and bottom-up cultural change initiatives (Rosenbluth et al., 2018; Keebler et al., 2022). Leadership must provide support to ensure transfer of training (Grossman and Salas, 2011), consistently message teamwork as a priority, and provide infrastructure for teaming events to take place, such as resources for handoff tool integration into electronic medical records (Abraham et al., 2021a, 2023; Michael et al., 2021). Resources and existing policies must be in place to provide trainees with opportunities to perform and reinforce newly trained skills (Tracey and Tews, 2005; Grossman and Salas, 2011). Additionally, there must be appraisal, recognition, and reward systems to incentivize trainees, faculty, and staff to use their acquired knowledge and skills (Tracey and Tews, 2005).

If training is delivered preclinically, learners transitioning to practice will disperse to different perioperative teams and units or different healthcare systems entirely, where policies and norms affecting culture vary greatly. Institutions that deliver teamwork training can implement initiatives to improve their own culture to support trained behaviors, but widespread dissemination of such initiatives is needed to support learners in external organizations. Therefore, until programs are appropriately scaled, the measurement of longitudinal impact will be limited to preclinical learners that transition to practice within the same institution.

## Discussion

Perioperative handoff interventions have become significantly more common in recent years; however, obstacles that limit provider adherence to these interventions threaten their sustainability and scalability. High-quality studies with sustainable interventions that demonstrate improved patient and provider outcomes remain elusive (Shahian et al., 2017; Abraham et al., 2021c; Burden et al., 2021; Riesenberg et al., 2022). Teamwork training is needed to enhance the sustainability of perioperative handoff interventions, and recent systematic reviews of perioperative teamwork training efforts call for robust, interprofessional programs that address the obstacles described above (Teunissen et al., 2020; Turcotte et al., 2022; Raveendran et al., 2023).

Many challenges in the perioperative environment affect patient handoffs, including interprofessional interdependence, handoff frequency, care acuity, time pressure, and environmental distractions (Etherington et al., 2019; Abraham et al., 2021b; Michael et al., 2021; Lazzara et al., 2022). Foundational teamwork training for all providers in perioperative handoffs will improve their ability to manage and overcome these challenges and, therefore, improve handoffs (Salas et al., 2008; Greilich et al., 2023). However, there are significant obstacles to delivering effective teamwork training, including barriers to facilitator-led education, appropriate timing and frequency of training delivery, delivery to an interprofessional audience, providing meaningful evaluation, and fostering conditions to sustain learned teamwork behaviors.

Addressing the complexities of teamwork in the perioperative environment poses an exciting opportunity to improve handoffs and meaningfully impact patient and provider outcomes. For example, though frequent handoffs create a challenge for teamwork, they also allow for repetitive practice and reflection on trained behaviors. This unique, high-need environment offers the ability to implement truly interprofessional interventions where miscommunications and poor coordination can cause life- and limb-threatening errors (The Joint Commission, 2017; Humphrey et al., 2022). The competencies acquired through effective teamwork training can be applied to handoffs as well as other critical teaming events in the perioperative environment, such as huddles, debriefs, and multidisciplinary rounds. Although the components discussed above present considerable obstacles to implementation, acknowledging their importance and discussing their associated challenges is the first step to building more meaningful, sustainable, and impactful teamwork training programs in the perioperative environment.

## Conclusion

Teamwork is critical to providing effective and reliable perioperative handoffs. Perioperative providers must be equipped with foundational teamwork competencies to improve team effectiveness, adherence to handoff interventions, and, ultimately, patient safety. To achieve sustained impact, user-centered training interventions must address the identified challenges of teamwork training in the perioperative environment.

## Data availability statement

The original contributions presented in the study are included in the article/supplementary material, further inquiries can be directed to the corresponding authors.

## Author contributions

SP and MK made significant contributions to the literature review, manuscript drafting, and revision. OH, JH, and AM provided critical reviews and revision guidance. PG and ES provided conceptual guidance and critical reviews of the manuscript. All authors have made substantial contributions to the conception, drafting, and revision of the manuscript and approved the final version for submission.

## Funding

The Quality Enhancement Plan: Team FIRST is an internally funded five-year program at UT Southwestern Medical Center for the fulfillment of an accreditation requirement of the Southern Association of Colleges and Schools.

## Conflict of interest

The authors declare that the research was conducted in the absence of any commercial or financial relationships that could be construed as a potential conflict of interest.

## Publisher’s note

All claims expressed in this article are solely those of the authors and do not necessarily represent those of their affiliated organizations, or those of the publisher, the editors and the reviewers. Any product that may be evaluated in this article, or claim that may be made by its manufacturer, is not guaranteed or endorsed by the publisher.
